# Progressive Multifocal Leukoencephalopathy Diagnosed by Metagenomic Next-Generation Sequencing of Cerebrospinal Fluid in an HIV Patient

**DOI:** 10.3389/fneur.2019.01202

**Published:** 2019-11-21

**Authors:** Huan Xia, Yuanlin Guan, Silvère D. Zaongo, Han Xia, Ziyu Wang, Zhongfang Yan, Ping Ma

**Affiliations:** ^1^Department of Infectious Disease, Tianjin Second People's Hospital, Tianjin, China; ^2^Hugobiotech Co., Ltd, Beijing, China

**Keywords:** progressive multifocal leukoencephalopathy, metagenomic next-generation sequencing, cerebrospinal fluid, HIV, PML

## Abstract

Metagenomic next-generation sequencing (mNGS) is a novel approach to identify pathogens undetected by conventional methods. Herein, we report a case in which mNGS was used to identify JC virus from the cerebrospinal fluid sample of an HIV positive patient with progressive multifocal leukoencephalopathy (PML).

## Background

Human immunodeficiency virus (HIV)-associated central nervous system (CNS) infections could be caused by any type of microbe (bacteria, viruses, fungi, parasites, and spirochetes) (1). Diagnosis of CNS infection remains a clinical challenge due to its protean presentation and imperfect diagnostic methods (2). At present, the typical laboratory diagnosis of CNS infection is based on routine technologies, such as smear, culture, serologic assays, and nucleic acid amplification tests. The detection rate of culture technique is low. The other assays broaden the range of detectable pathogens, but prior knowledge is essential; these methods also usually do not permit the discovery of novel or unforeseen pathogens (3). Identification of causative pathogens is vital for the accurate diagnosis and could be needed for the timely introduction of targeted therapy. A method which has been recently applied to pathogen detection in cases of CNS infection is metagenomic next-generation sequencing (mNGS) (2, 4). mNGS provides an unbiased approach which allows universal pathogen detection from clinical specimens, and it is particularly suitable for novel organism discovery (3). Herein, we report a case of progressive multifocal leukoencephalopathy (PML) detected by mNGS in the cerebrospinal fluid (CSF) sample of an HIV-1 positive patient.

## Case Presentation

**Figure 1:**
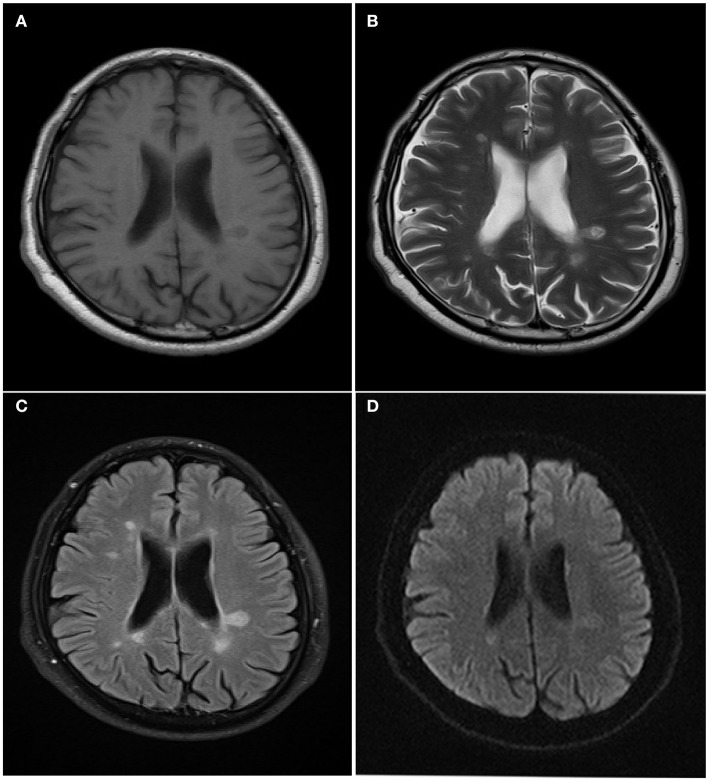
Magnetic resonance imaging results. T1-weighted images **(A)** showed multiple oval hypointense lesions in the white matter arround bilateral. These lesions were hyperintense on T2-weighted images **(B)** and fluid-attenuated inversion recovery images **(C)**. Diffusion-weighted images (DWI) demonstrated several increased signal intensity lesions in the white matter arround bilateral ventricles **(D)**.

A 29-year-old woman with dizziness and limb weakness for 2 weeks was admitted to the department of neurology of another hospital. There, parts of examination including brain CT,MRI, and blood tests were done. She was subsequently tested positive for HIV-1 virus infection (CD4 = 67/mm^3^; Viral Load = 2.66 × 10^5^ copies/mL) and transferred to our hospital. MRI of the brain showed multiple lesions more marked in the white matter around bilateral ventriculus cerebri lateralis (Figures 1A–D). Physical examination demonstrated no abnormalities. Neurological examination did not reveal any significant abnormality. Finger-to-nose and heel-to-knee tests demonstrated no abnormalities. Bilateral deep tendon reflexes were normal and Babinski reflex was also normal. However, we noted that the patient was somewhat slow in reaction. Blood testing showed no sign of bacterial, *Mycobacterium*, and fungal infection.

We quickly suspected PML but the PCR technique for JC virus detection in CSF is not available in our hospital. CSF analysis were within the normality range (glucose, cells, and CSF-serum albumin ratio), but the protein content was significantly increased to 0.6 g/L (normal range, 0.2–0.4 g/L). CSF cryptococcal antigen, *Treponema pallidum* particle agglutination assay, culture were negative. India ink staining and acid-fast staining of CSF were also negative. Some of the patient's CSF sample were collected and cryopreserved. Polymerase chain reaction (PCR) performed from the CSF sample for CMV, HSV, and EBV identification was negative. We failed to find another public or private lab to perform the JC virus PCR test in Tianjin, China. Thus, for a quick diagnosis, we had no choice but to send the CSF for NGS analysis to an independent clinical lab (KingMed Diagnostics Group Co., Ltd). An unbiased metagenomic sequencing of CSF revealed a high load of *human polyomavirus 2* [also known as JC virus (5)]. Based on the results of clinical features, imaging findings, and mNGS testing; the diagnosis of HIV-related PML was finally established. The patient was, therefore, taking up antiretroviral therapy (ART) (dolutegravir-abacavir-lamivudine, one tablet a day). On day 6 of her hospitalization, we found a lab in Beijing (Peking Union Medical College Hospital) where JC virus PCR is performed for research. We, therefore, sent a cryopreserved CSF sample there for analysis. However, the JC virus PCR was negative (limit of detection estimated at 500 copies/mL). Two months after her admission, HIV virus RNA became undetectable (20 copies/mL) and CD4 count increased from 67 to 266/mm^3^. However, we noted that her neurological symptoms were worsening after initiation of the effective ART. The clinical features of our patient showed a progressive worsening in the followed months. Indeed, she developed new symptoms of headache, generalized fatigue, progressive generalized slowness, speech difficulty, and presented decline altered mental status. The neurologic examination revealed mild memory and executive dysfunction, mild lower-limb ataxia. The course was consistent with PML-immune reconstitution inflammatory syndromes. Five months later she was alive with a deteriorating neurological status. An MRI and lumbar puncture were not repeated as the family refused to allow additional tests.

## Metagenomic Next-Generation Sequencing

**Figure 2:**
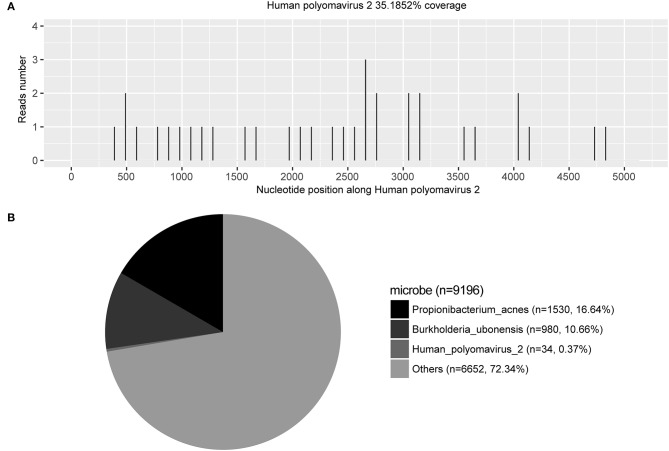
Pathogen identification from cerebrospinal fluid sample using next-generation sequencing method. **(A)** The number of sequencing reads identified corresponding to *Human polyomavirus 2* [also known as JC virus (5)] was 34; with genome coverage 35.1825%. **(B)** Reads distribution of total DNA sequence in the CSF sample without human host.

**Figure 3:**
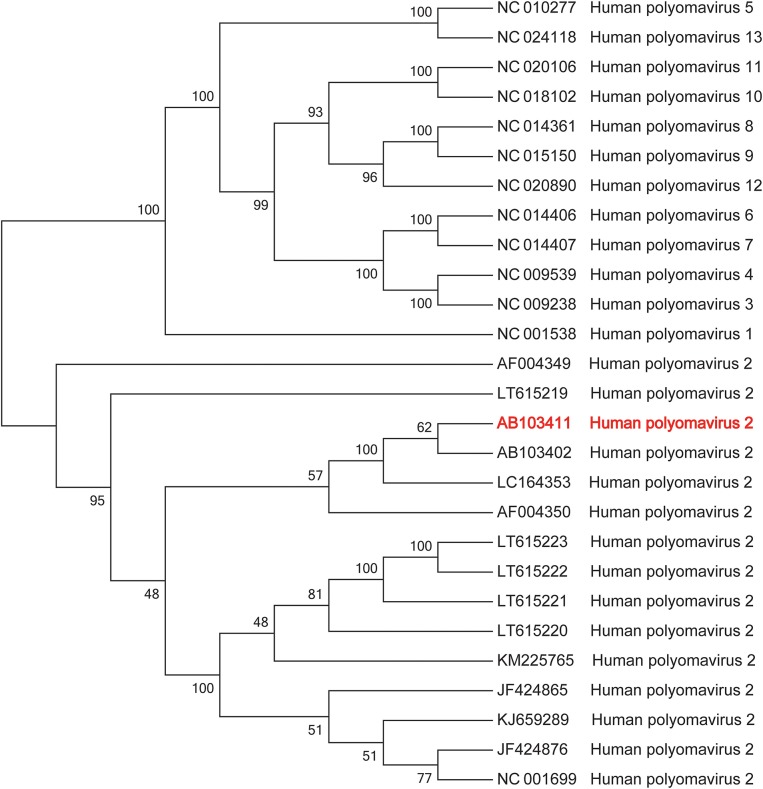
Maximum likelihood phylogenetic tree of *Human polyomavirus*. *Human polyomavirus 2* AB103411 (in red) was the most similar strain in the cerebrospinal fluid of the patient.

**Figure 4:**
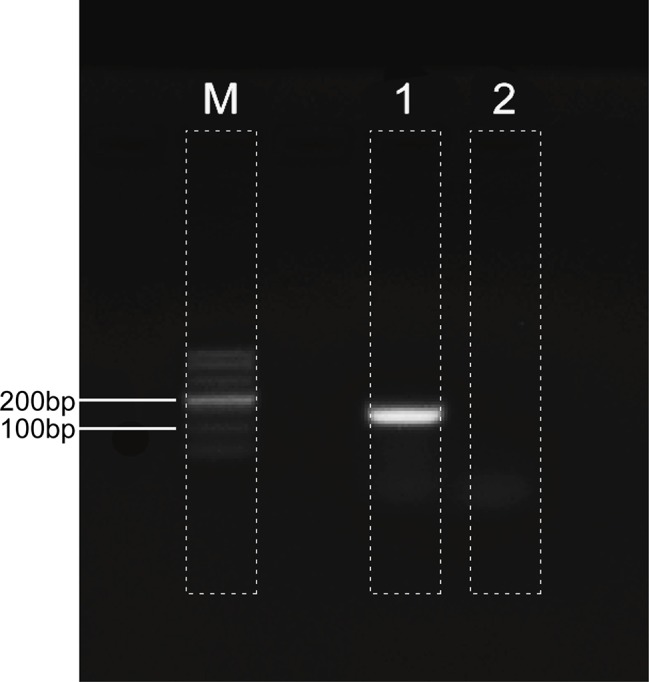
*Human polyomavirus 2* detection by PCR. Lane 1: 129-bp PCR product of the primer: F-GGTTTAGGCCAGTTGCTGACTT, R-GTCTCCCCATACCAACATTAGCTT; Lane M: DNA ladder. Lane 2: Negative control.

The patient's CSF specimen was analyzed by metagenomic sequencing. DNA was extracted from 200 μL of CSF specimen using a TIANamp Micro DNA Kit DP316. The sequencing library was constructed via NEBNext Ultra II DNA library Prep Kit. The library concentration and quality were checked using Qubit and agarose gel electrophoresis. The library was sequenced by Illumina Miniseq system. The raw data were generated by Illumina sequencing machine. High-quality data were generated after filtering out adapter, low-quality, low-complexity, and shorter reads. Then, the human sequences were excluded by mapping reads to the human reference genome via SNAP tool. The remaining data were aligned to the microbial genome database, which is more than 20000 genome. Finally, we got the microbial compositions of the sample. Thus, we noted 34 sequencing reads uniquely aligned to *Human polyomavirus 2* genome (Figure 2B), and these reads enclosed a high coverage percentage (35.18%) of *Human polyomavirus 2* genome (Figure 2A). We, therefore, named the virus identified as *Human polyomavirus 2* (GenBank ID: AB103411). The phylogenetic tree presenting our sequence similarities with other Human polyomavirus (1–13) is shown in Figure 3. To validate the results of mNGS, sequence-specific PCR identification of *Human polyomavirus 2* with a target fragment was carried out using the primers F-GGTTTAGGCCAGTTGCTGACTT and R-GTCTCCCCATACCAACATTAGCTT. The PCR products (Figure 4) were sequenced and then mapped to the nucleotide database with the online NCBI blast (https://blast.ncbi.nlm.nih.gov/Blast.cgi). A 129 bp PCR product was assembled and found to be 100% identical to a reference *Human polyomavirus 2* sequence. Consequently, all these results indicated that the patient was infected with *Human polyomavirus 2*.

## Discussion

PML is a demyelinating disease of the CNS caused by reactivation of latent JC virus that typically infects oligodendrocytes and astrocytes (6, 7), and remains one of the most common CNS opportunistic infections that often occur in HIV patients with low CD4+ cell count (< 200 cells/μL) (1). The definitive diagnosis of PML could be made based on clinical features and radiographic findings coupled with CSF or brain PCR detection for JC virus (8–10). Ascertainment of the disease represents a major challenge in the clinical setting, PML could be misdiagnosed as HIV encephalopathy, acute demyelinating encephalomyeilitis, and primary CNS lymphoma on account of the predominant involvement of the brain white matter (9). In regards to our patient, the clinical presentation was atypical and non-specific. Moreover, brain imaging features were not specific enough to accurately make a precise diagnosis.

Sometimes when clinical suspicion remains and yet non-invasive neuroimagings are equivocal; laboratory findings mostly enable the correct diagnosis. Unfortunately, routine technology has limited power. Under diagnosis due to a shortage of imaging or diagnostic methods are common in developing countries including China (11). Quantitative PCR techniques in CSF has become the cornerstone of PML diagnosis. In fact, its specificity for JCV is 98%, but the sensitivity is only 58–75% (9, 12), and the test can often be negative during the early phase of the disease (13). Hence, a negative PCR does not rule out PML (9). It is vital to ensure that the laboratory diagnostic technique can detect JCV-DNA at very low-level of copy numbers (9, 13). In addition, HIV patients with PML due to low or undetectable JCV copy number have been described with increased frequency (12, 14). Yet in China, few laboratories could perform JCV detection in CSF using PCR, and some detection limit are as high as 500 copies/mL; such assays normally rely on prior suspicion of JCV infection. Indeed, the PCR technique could be an easier and a cheaper alternative. But in our context, mNGS was the best alternative as it quickly oriented the diagnostic.

Herein, we described the metagenomic sequencing analysis as a diagnostic tool to establish the presence of JCV infection in the CSF sample and finally confirmed the etiology of the brain lesions. In this patient, conventional diagnostic studies had failed to define causative pathogens. Our patient represents a good proof that mNGS studies are useful and perhaps provide a better characterization of the JC virus genome for diagnosis purpose.

To our knowledge, there are no reports of mNGS used for PML detection from CSF. There is only one published PML case diagnosed by mNGS from a brain biopsy. RNA reads aligned to JCV and the result was confirmed by immunohistochemistry and neuropathology (4). In our patient, the demonstration of JC viral DNA through mNGS coupled with the appropriate clinical and radiologic features was sufficient to conclude on the PML diagnosis. Thus, we suggest that brain biopsy could be avoided.

We did not verified the sensitivity and specificity of mNGS technique for JC virus detection. But, We evaluated the sensitivity and specificity of mNGS for virus detection through spike-in experiments with other virus. The sensitivity was > 95% and the specificity was > 99%. The limit of virus detection for mNGS was estimated at 10 copies/mL. We have also detected JC virus by mNGS in several other specimens of immunocompromised patients. In our case, we detected 34 JC virus reads with mNGS. And all these sequences only matched to the genome of JC virus (identity > 98%). But the alignment with other genome sequences was very poor (identity < 90%), indicating that the JC virus sequences we detected had a good specificity.

Sometimes mNGS findings were considered indeterminate as suspected agents, frequently seen as contaminants for their pathogenic role, could not be testified by cultures or other techniques (15, 16). The results obtained from certain specimens could critically be impacted by contamination. This is particularly relevant for the mNGS analysis of CSF which is normally sterile. Common reagent contaminants in CSF samples include members of *Enterobacteriaceae* or *Cutibaterium acnes*, but so far *Mycobacterium tuberculosis* or JC virus are not found as contaminants in reagents or part of normal microbiota (17). Accordingly, in our study, there is no need to further interpret the mNGS results.

The turnaround time for mNGS in our case was 4 days from clinical specimen receipt. With this technique, a timely etiologic diagnosis could be attained. Furthermore, it could help to narrow antimicrobial therapy and consequently reduce the treatment cost and drug-related toxicities. mNGS studies of CSF provide possibility for great improvements in our ability to detect a broad scope of CNS infection agents, with potential benefits in sensitivity, speed, and cost. It might supplement the currently used diagnostic methods for the determination of CNS disorders. With suitable case selection, proper sample managing, and result interpretation; mNGS could emerge as a promising methodology for infectious disease diagnostics. It really is expected that over another couple of years, the cost and time-to-result of metagenomics will certainly reduce, and with this, it is foreseen that it will be possible to offer this technique as the first-line diagnostic check.

## Ethics Statement

The studies involving human participants were reviewed and approved by Tianjin Second People's Hospital Research Ethics Committee. The patients/participants provided their written informed consent to participate in this study. Written consent for publication was obtained from the patient's family.

## Author Contributions

ZW, HuX, ZY, and PM were involved in the clinical management of this patient. HaX and YG performed the mNGS and human polyomavirus 2-specific PCR. HuX, HaX, SZ, YG, and PM wrote the article. All authors read and approved the final manuscript.

### Conflict of Interest

The authors declare that the research was conducted in the absence of any commercial or financial relationships that could be construed as a potential conflict of interest.
